# Differential effects of body composition on left ventricular geometric remodelling and aortic elastic dysfunction in obesity

**DOI:** 10.1186/1532-429X-18-S1-Q38

**Published:** 2016-01-27

**Authors:** Jennifer J Rayner, Rajarshi Banerjee, Jane M Francis, Stefan Neubauer, Oliver Rider

**Affiliations:** grid.4991.50000000419368948University of Oxford, Oxford, United Kingdom

## Background

Although obesity per se has been linked to increased left ventricular (LV) mass, and concentric LV remodelling, the effect of individual variation in body composition and fat distribution is poorly understood. However this is likely to be an important determinant of adverse cardiovascular remodelling. Our aim was to use cardiovascular magnetic resonance (CMR) and dual-energy X-Ray absorptiometry (DEXA) to investigate the relationship between body composition and cardiovascular remodelling in obesity.

## Methods

133 subjects (male n = 45) across a wide range of body mass index (BMI 18.5-59.2 kg/m^2^) with no identifiable cardiac risk factors (average systolic blood pressure (SBP) 118 ± 11, diastolic blood pressure (DBP) 74 ± 8 mmHg, glucose 5.0 ± 0.5 mmol/l, cholesterol 5.0 ± 0.8 mmol/l) underwent DEXA for body composition (lean mass, total fat mass), MRI assessment (1.5T) for abdominal visceral fat area and CMR for LV geometry (mass and mass:volume ratio). Aortic distensibility was also determined as the average of 3 levels; the ascending and proximal descending aorta at pulmonary artery level, and the abdominal aorta.

## Results

Total LV mass was correlated positively with lean body mass (r 0.87, p < 0.001), visceral fat (r 0.55, p < 0.001), insulin (r 0.44, p < 0.001), SBP (r 0.38, <0.001), and total fat mass (r 0.32, p < 0.001). Multiple regression of these variables showed only lean mass (b = +2.3, p < 0.001) and SBP (b = +0.3, p = 0.03) were independent predictors of total LV mass. This suggests lean mass rather than fat mass increase with obesity is the major determinant of total LV weight (R^2^ of model 0.77, p < .0001). In contrast, although lean mass (r 0.72, p < 0.001) and total fat mass (r 0.62, p < 0.001) were correlated with concentric LV remodelling, multiple regression showed only visceral fat to be predictive of concentric LV remodelling (b = +0.001, p < 0.03). This suggests that it is the distribution of fat within the visceral depot that is more important than the total fat volume in generating concentric LV remodelling in obesity. Although aortic distensibility was correlated with age (r -0.65, p < 0.001), visceral fat mass (r -0.45, p < 0.001), SBP (r -0.52, p < 0.001), total fat mass (r -0.36, p = 0.007) and lean mass (r -0.20, p = 0.03), multiple regression showed that only age (b = -0.13, p < 0.001), SBP (b = -0.04, p = 0.01) and visceral fat were independent predictors (R^2^ of model 0.59, p < .0001). Aortic stiffness showed a similar pattern to concentric LV remodelling, suggesting that visceral fat area is more important than total adiposity in reducing aortic elastic function.

## Conclusions

Body composition plays an important role in the pattern of cardiovascular remodelling in obesity. Interestingly, the increase in lean body mass rather than fat mass that accompanies obesity appears to be the main driver behind total LV mass increase. In contrast, it is abdominal visceral fat that is more predictive of concentric LV remodelling and increased aortic stiffness.

**Figure 1:**
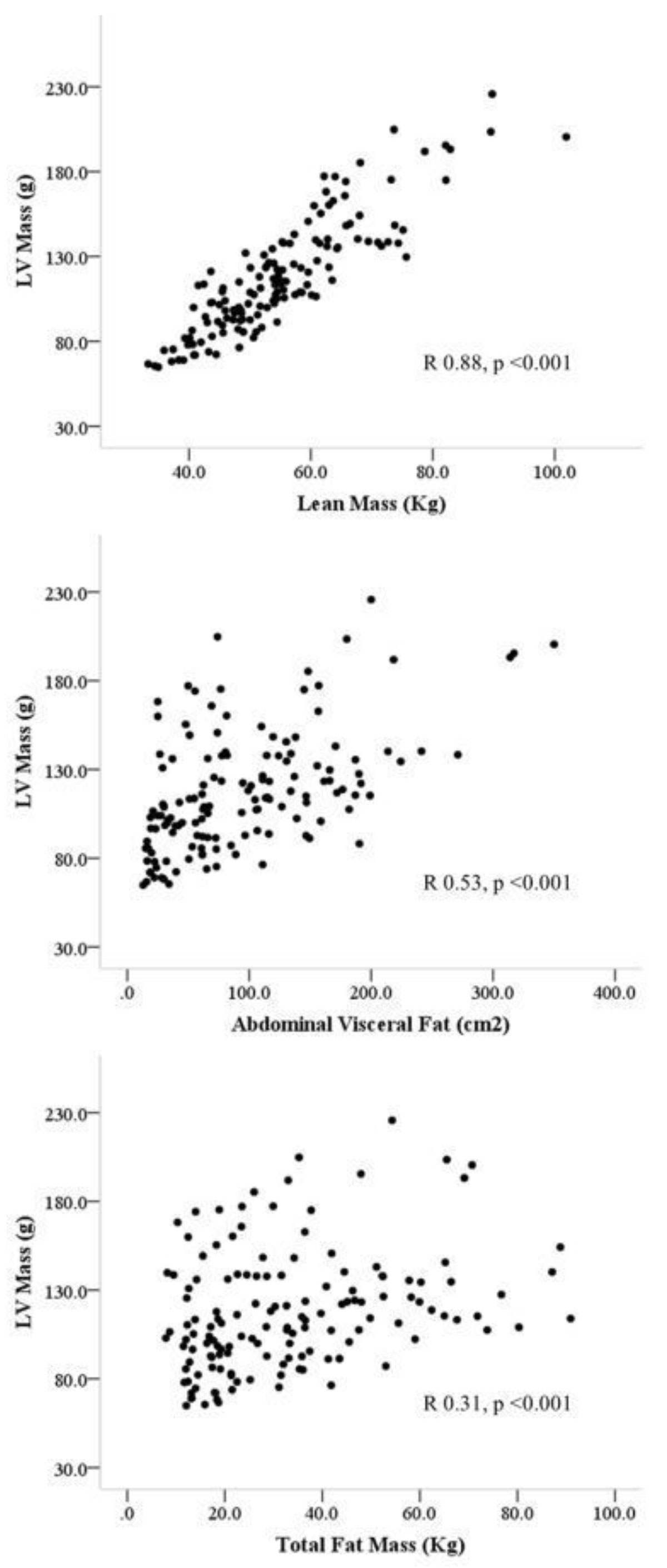
**The larger effect of lean mass (upper panel) than visceral fat (middle panel) or total fat (lower panel) in determining LV mass in obesity**.

